# IL4I1 binds to TMPRSS13 and competes with SARS-CoV-2 spike

**DOI:** 10.3389/fimmu.2022.982839

**Published:** 2022-09-05

**Authors:** Jérôme Gatineau, Charlotte Nidercorne, Aurélie Dupont, Marie-Line Puiffe, José L. Cohen, Valérie Molinier-Frenkel, Florence Niedergang, Flavia Castellano

**Affiliations:** ^1^ Univ Paris Est Creteil, INSERM, IMRB, Creteil, France; ^2^ Université Paris Cité, CNRS, INSERM, Institut Cochin, CNRS, Paris, France; ^3^ AP-HP, Hopital H Mondor, CIC Biotherapies, Créteil, France; ^4^ AP-HP, Hopital Henri Mondor, Departement d’Hematologie-Immunologie, Créteil, France; ^5^ AP-HP, Hopital Henri Mondor, Plateforme des Ressources Biologiques, Créteil, France

**Keywords:** IL4I1, immunosuppressive enzymes, SARS-CoV-2, spike, cancer therapy, moonlighting protein

## Abstract

The secreted enzyme interleukin four-induced gene 1 (IL4I1) is involved in the negative control of the adaptive immune response. IL4I1 expression in human cancer is frequent and correlates with poor survival and resistance to immunotherapy. Nevertheless, its mechanism of action remains partially unknown. Here, we identified transmembrane serine protease 13 (TMPRSS13) as an immune cell-expressed surface protein that binds IL4I1. TMPRSS13 is a paralog of TMPRSS2, of which the protease activity participates in the cleavage of SARS-CoV-2 spike protein and facilitates virus induced-membrane fusion. We show that TMPRSS13 is expressed by human lymphocytes, monocytes and monocyte-derived macrophages, can cleave the spike protein and allow SARS-CoV-2 spike pseudotyped virus entry into cells. We identify regions of homology between IL4I1 and spike and demonstrate competition between the two proteins for TMPRSS13 binding. These findings may be relevant for both interfering with SARS-CoV-2 infection and limiting IL4I1-dependent immunosuppressive activity in cancer.

## Introduction

Negative control of the immune response is achieved through multiple mechanisms, amongst which expression of the enzyme interleukin four-induced gene 1 (IL4I1). IL4I1 is a secreted L-amino acid oxidase that catabolizes the essential amino acid phenylalanine and, to a lesser extent tryptophan and arginine, into the corresponding α-keto acids, H_2_O_2_, and NH_3_ (1). Antigen-presenting cells are the main producers of IL4I1 under physiological conditions (2, 3). IL4I1 regulates B and T-cell activation and proliferation (3–5) and facilitates the differentiation of FoxP3^+^ regulatory T cells from naïve CD4^+^ T cells (6). In the context of cancer, IL4I1 is expressed by infiltrating macrophages and occasionally by the tumor cells (7). In mouse tumor models, IL4I1 facilitates escape from the immune response (8) and in humans, its local expression correlates with decreased survival and a pejorative outcome (9–11).

The mechanisms of action of IL4I1 are only partially known. The rapid IL4I1-mediated inhibitory effect on TCR signaling, within minutes from T-cell activation, its focal secretion into the synaptic cleft, and its detection on the T-cell membrane (5), suggest the existence of an IL4I1 receptor.

Patients with severe Coronavirus disease 19 (COVID-19), caused by severe acute respiratory syndrome Coronavirus 2 (SARS-CoV-2), present profound dysregulation of the immune response, characterized by hyper-inflammation (12) and lymphopenia (13). Virus entry into cells depends on binding of the spike protein to cellular receptor angiotensin converting enzyme 2 (ACE2) and on its priming by host cell transmembrane serine protease (TMPRSS) 2 (14, 15). Spike cleavage leads to the liberation of an S1 subunit that contains the receptor-binding domain, while exposing the S2 fragment, which allows fusion of the viral envelop with the cell membrane. The TMPRSS proteins are type II transmembrane serine proteases. Activation of the zymogen requires the cleavage of the C-terminal extracellular part of the protein, which remains attached to the cell surface through intramolecular disulfide bonds (16). Other proteases of this family have been shown to activate spike-pseudotyped virus (17).

Here, we searched for an IL4I1 receptor at the surface of immune cells and identified TMPRSS13, a poorly known paralogue of TMPRSS2. This protease can substitute for TMPRSS2, allowing infection of human monocytes-derived macrophages (hMDM) by spike-pseudotyped virus (LVS). Most interestingly, IL4I1 and SARS-CoV-2 spike share regions of homology and can compete for TMPRSS13 binding.

## Results

### Identification of TMPRSS13 as an IL4I1 receptor

**Figure 1:**
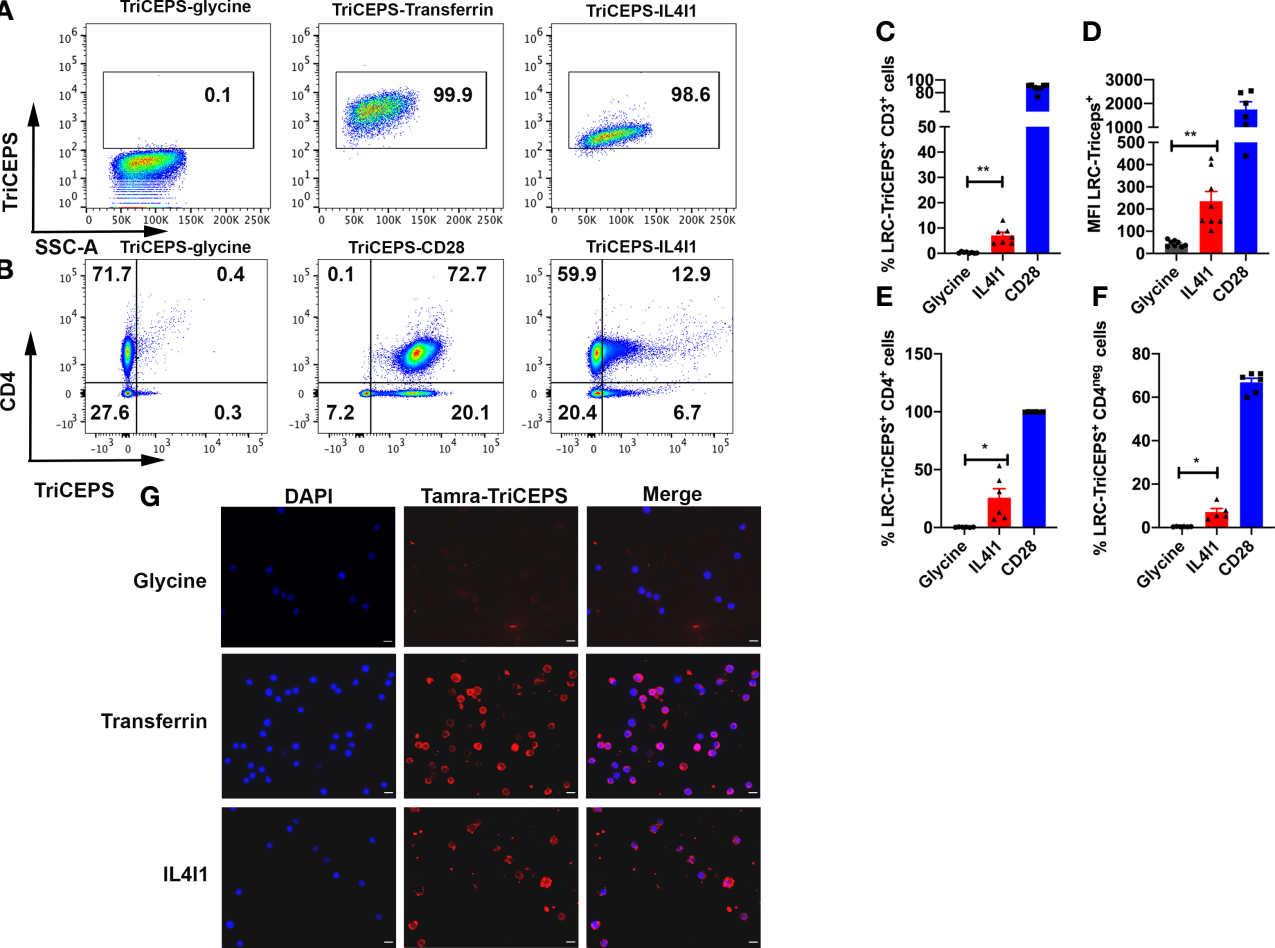
IL4I1 binds to Jurkat cells and human primary T lymphocytes. **(A)** Jurkat cells were incubated with TriCEPS-glycine, TriCEPS-transferrin, or TriCEPS-IL4I1 at 4°C for 1 h **(B)** Purified CD3^+^ primary T cells were similarly treated (TriCEPS-anti-CD28 antibodies were used instead of TriCEPS-transferrin). After binding, cells were labeled with a viability dye and streptavidin to reveal bound TriCEPS-conjugates. For T lymphocytes, cells were also labeled with an anti-CD4 antibody before analysis by FCM. Percentage of TriCEPS-positive CD3 lymphocytes **(C)**, mean intensity of fluorescence (MFI) of TriCEPS on CD3^+^ lymphocytes **(D)**, percentage of TriCEPS-positive CD4^+^ T cells **(E)**, and TriCEPS-positive CD4^-^ T cells **(F)**. Data are from N= 4 independent experiments for Jurkat cells and N = 3 for CD4^+^. **(G)** Visualization by IF of IL4I1 binding using TAMRATriCEPS. Jurkat cells were incubated with TAMRA-TriCEPS bound to glycine (top), transferrin (middle), or IL4I1 (bottom). Nuclei were labeled with DAPI. Data are representative from N =3 independent experiments. Bar = 10μm. Statistical analysis with a two-tailed paired t test. *p < 0.05, **p < 0.01.

We used the ligand receptor capture (LRC)-TriCEPS^®^ technique (18) to identify the protein(s) responsible for IL4I1 binding to the surface of T lymphocytes. We conjugated the TriCEPS reagent with a positive control (transferrin for Jurkat T cells or anti-CD28 antibodies for primary T lymphocytes), a negative control (glycine), or recombinant human IL4I1. We next incubated these conjugates at 4°C with the Jurkat cells (**Figure 1A**) or sorted primary CD3^+^ lymphocytes (**Figure 1B**) and revealed the cell-surface-bound conjugates by flow cytometry (FCM) after TriCEPS staining (gating strategy **Figure S1**). No TriCEPS-glycine bound to Jurkat cells, whereas almost all the cells were labeled with TriCEPS-transferrin. In accordance with our previous observations (5), TriCEPS-IL4I1 bound to Jurkat cells. The MFI of TriCEPS-IL4I1 was lower than that of TriCEPS-transferrin (432 ± 110.00 and 3888.3 ± 1439.2 respectively), indicating a lower level of expression of the IL4I1 receptor than that of the transferrin receptor. When we tested primary CD3^+^ T lymphocytes, ~90% percent of the CD3^+^ population bound the TriCEPS-CD28 conjugate (**Figures 1B–F**). A fraction of both CD4^+^ and CD4^-^ CD3^+^ cells (25.7% ± 1.4 and 7.2% ± 0.3 respectively) bound TriCEPS-IL4I1, confirming a specific surface interaction of IL4I1 with T lymphocytes. We further confirmed IL4I1 binding using TAMRA-labeled TriCEPS and immunofluorescence microscopy (IF) (**Figure 1G**). Conjugates were visible at the cell surface for both transferrin and IL4I1, whereas we detected no signal with glycine. In accordance with the FCM results, the labeling was more intense for transferrin than for IL4I1. Thus, IL4I1 binds specifically to T lymphocytes.

**Figure 2:**
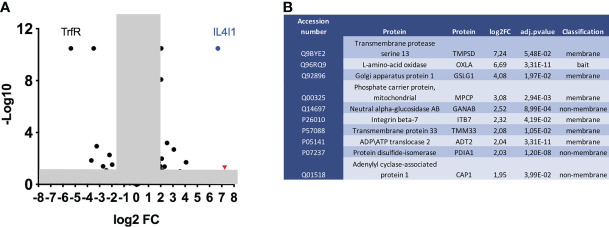
Identification of potential IL4I1 receptor candidates by TriCEPS/LC-MS. Jurkat cells were incubated with TriCEPS-transferrin or TriCEPS-IL4I1 followed by LC/MS. **(A)** Comparative data are shown as a volcano plot, with p < 0.05 and the fold change (log2 FC) > 2 outside the shaded box. Data were derived from three independent technical replicates. TMPRSS13 red triangle, IL4I1 blue dot. **(B)** List of the identified proteins by TriCEPS-IL4I1 capture.

We next sought to identify the protein(s) implicated in IL4I1 binding. We cross-linked the TriCEPS-IL4I1 conjugate to the Jurkat cell surface by exposing the cells to gentle oxidizing conditions with sodium-metaperiodate. TriCEPS-transferrin was used as a positive control. Cells were then lysed and analyzed by liquid chromatography/mass spectrometry (LC-MS) (**Figures 2A, B**). Mass spectrometry analysis led to the identification of 163 differentially present proteins. Twenty-one proteins in the samples incubated with TriCEPS-IL4I1, showed a Log2 fold change (FC) of 2 or more, including IL4I1 itself. However, aside from IL4I1, only nine were significantly enriched. Amongst them, the serine transmembrane protease TMPRSS13 showed a FC of 7.2, similar to the enrichment of the IL4I1 bait (Log2 FC of 6.7). We thus focused on this protein for subsequent analysis.

We cloned the TMPRSS13 cDNA and stably expressed a DYK-tagged protein in HEK cells (**Figure S2**) and chose a strong expressing clone (HEK-T) verified by western blotting (WB) and IF using an anti-tag antibody. The recombinant protein showed an apparent molecular weight of approximately 70 kDa (**Figures S2A, B**), as already described (19). We detected other bands using an anti-TMPRSS13 antibody that recognizes the C-terminal part of the protein on transiently transfected cells (**Figure S2C**). These bands likely correspond to intermediate products of glycosylation or cleavage of the extracellular C-terminus of the protein, as described by Murray et al. (19). TMPRSS13 and the closely related TMPRSS2 protein are not expressed by the lung adenocarcinoma cell line A549 (20). Thus, we used A549 cells overexpressing or not TMPRSS2 to test the specificity of our anti-TMPRSS13 antibody. Although two faint bands were detected at molecular weights expected for TMPRSS2, their intensity was similar in both cell lines, indicating that the antibody did not recognize TMPRSS2 (**Figure S2D**). Moreover, the anti-TMPRSS13 antibody could detect the protein at the cell surface in FCM analyses (**Figure S2E**). Finally, the IF labeling of the HEK-T cells with anti-tag (DYK) and with the commercially available anti-TMPRSS13 antibody directed towards the C-terminus of TMPRSS13 showed similar patterns (**Figures S2F–I**), allowing us to validate this antibody for the study of cells naturally expressing TMPRSS13.

**Figure 3:**
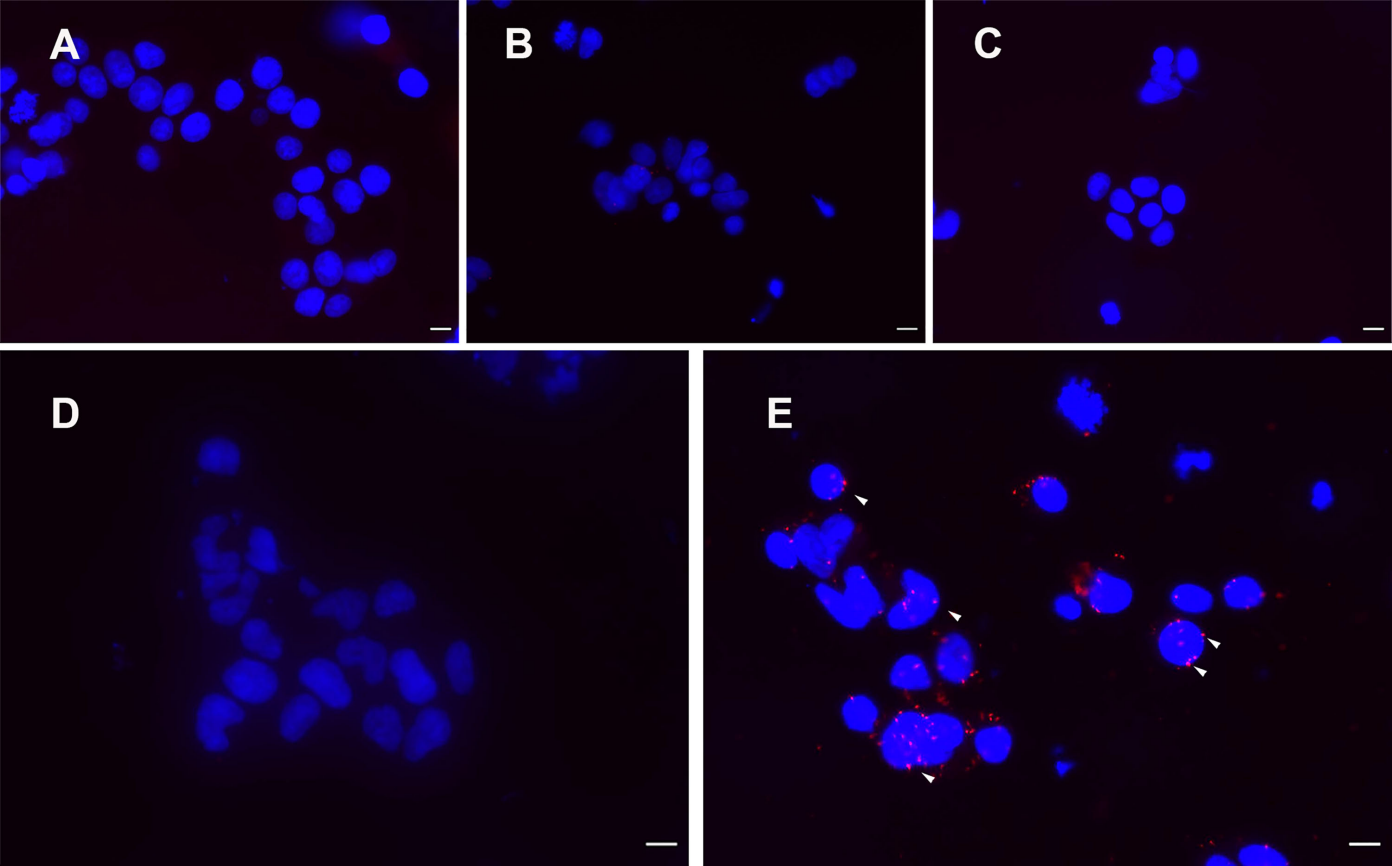
Proximity ligation assay reveals spatial proximity of IL4I1 and TMPRSS13. HEK or HEK-T cells were incubated with recombinant hIL4I1 at 4°C for 1 h and tested by the PLA. Nuclei were stained with DAPI. **(A)** No primary antibodies, **(B)** primary anti-DYK antibody only, **(C)** primary anti-IL4I1 antibody only, **(D)** HEK cells incubated with IL4I1 and primary and secondary antibodies, **(E)** HEK-T cells incubated with IL4I1 and primary and secondary antibodies. Data are representative from N =3 independent experiments. Bar = 10 μM. White arrowheads indicate some of the PLA interaction signals.

To confirm the interaction of IL4I1 with TMPRSS13 on HEK-T and Jurkat cells, we first performed the proximity ligation assay (PLA). HEK and HEK-T cells were incubated with recombinant IL4I1 and processed for PLA using a rabbit primary antibody against IL4I1 and a mouse antibody directed against the DYK tag of recombinant TMPRSS13. No positive signal was detectable in the experimental controls nor in HEK cells incubated with IL4I1, whereas a strong PLA-positive signal was visible on the HEK-T cells incubated with the protein, indicating proximity between IL4I1 and TMPRSS13 **(** **Figure 3**). On average, the cells showed 4.1 ± 2.4 dots per cell. A similar signal was detected on Jurkat cells but with fewer spots per cell (1.3 ± 0.4) (**Figure S3**).

**Figure 4:**
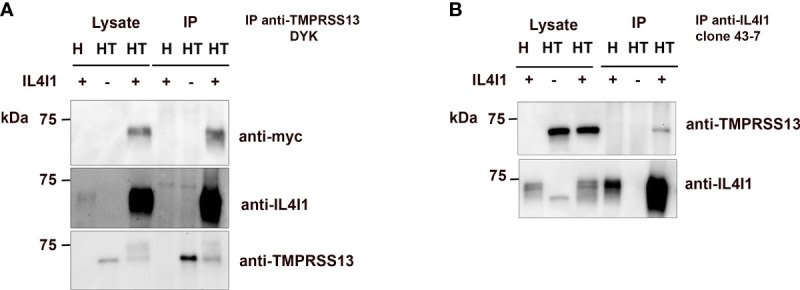
IL4I1 and TMPRSS13 co-immunoprecipitate. Concentrated Myc-tagged IL4I1 was added to wells containing HEK or HEK-T cells and incubated for 1 h at 4 °C. Cells were lysed and **(A)** immunoprecipitated with anti-DYK antibody (for TMPRSS13) and the precipitates analyzed by WB using the indicated antibodies. **(B)** Reverse co-IP of **(A)** performed with anti-IL4I1 antibodies. Data are representative from N>3 independent experiments.

As PLA indicates only the proximity of IL4I1 and TMPRSS13, we further validated the interaction by performing co-immunoprecipitation (Co-IP) experiments (**Figure 4**). After incubation with IL4I1, HEK control cells and HEK-T cells were lysed and TMPRSS13 (**Figure 4A**) or IL4I1 (**Figure 4B**) precipitated, respectively. IL4I1 was co-immunoprecipitated with TMPRSS13 (**Figure 4A**), as revealed using either an anti-tag (Myc) or anti-IL4I1 antibody. We observed no precipitation when TMPRSS13 was not expressed. In the reverse Co-IP, we specifically detected TMPRSS13 after precipitation of IL4I1 in HEK-T cells only (**Figure 4B**). Thus, IL4I1 and TMPRSS13 directly interact with each other and TMPRSS13 may represent an IL4I1-receptor at the surface of T cells.

### Expression of TMPRSS13 by immune cells

**Figure 5:**
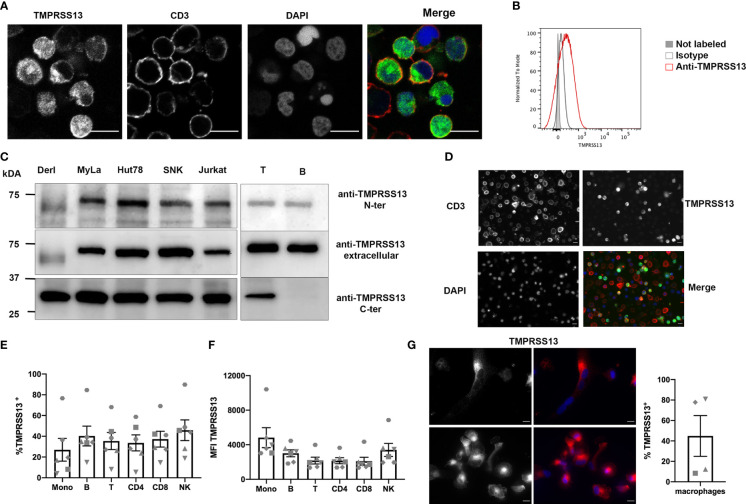
Expression of TMPRSS13 by Jurkat cells and human circulating immune cells. **(A)** Immunofluorescence surface labeling of Jurkat cells with anti-TMPRSS13 (C-ter) (green) and anti-CD3 (red) antibodies. Nuclei were stained with DAPI. Bar = 10 μM. **(B)** Flow cytometry of Jurkat cells using an anti-TMPRSS13 (C-ter) antibody. Filled grey plot: non-labeled cells, gray line: isotype control, red line: TMPRSS13 staining. **(C)** Western blotting of TMPRSS13 expression in Derl, Myla, HuT78, SNK, Jurkat cells, purified CD3 lymphocytes, and purified B lymphocytes using anti-TMPRSS13 antibodies directed either to the C-terminal domain (C-ter), the entire extracellular domain, or the N-terminal domain (N-ter) of the protein. **(D)** Immunofluorescence surface labeling of PBMCs from healthy donors with anti-TMPRSS13 (green) and anti-CD3 (red) antibodies. Nuclei were stained with DAPI. Bar = 10μM. **(E, F)** Flow cytometry of TMPRSS13 of PBMCs labeled with Fixable Viability Dye (VD) and stained with anti-CD3-PE, anti-CD4-BV510, anti-CD8-BV711, anti CD14-FITC, anti-CD19-PC7, and anti-CD56-PC5.5. After fixation, cells were stained for TMPRSS13. Data were analyzed on the various populations after the exclusion of VD-positive cells. **(E)** Percentage of TMPRSS13-positive cells in each subpopulation. **(F)** MFI. Data are from the analysis of N = 6 healthy donors. **(G)** Expression of TMPRSS13 by hMDM. Left: IF with anti-TMPRSS13 antibody of hMDM. Bar = 10 μM. Right: FCM of TMPRSS13 in hMDM. Data are from N = 4 healthy donors.

Little information is available concerning physiological TMPRSS13 protein expression, but RT-PCR data indicate that it may be produced by immune cells (21). Thus, we verified TMPRSS13 expression by labeling Jurkat cells with the validated anti-TMPRSS13 antibody (**Figure 5**). The Jurkat cells expressed TMPRSS13 at the cell surface, as visualized by IF (**Figure 5A**) and FCM (**Figure 5B**). We next analyzed TMPRSS13 expression by WB on several T-cell lines, including Jurkat cells, and on sorted primary human CD3^+^ and CD20^+^ lymphocytes using the validated antibody, which is directed towards the C-terminus, and two different commercial antibodies targeting N-terminal epitopes (**Figure 5C**). Both antibodies targeting the N-terminal epitopes revealed a protein of approximately 70 kDa in all lysates, corresponding to the molecular weight of the full-length TMPRSS13. However, when the C-terminus-directed antibody was used, the full-length protein was barely visible. By contrast, a strong signal at approximately 30 kDa was revealed in all T-cell lines and primary T cells but was absent from B cells. The molecular weight of this fragment corresponds to that of the C-terminal catalytic domain (19). We next analyzed TMPRSS13 expression on human peripheral blood mononuclear cells (PBMCs) by IF (**Figure 5D**). Some T cells were TMPRSS13^+^ and showed fainter signals then larger CD3^-^ cells, possibly monocytes. We refined our analysis by FCM (**Figures 5E, F**) by measuring TMPRSS13 expression in CD4^+^ and CD8^+^ T cell subsets, B cells, monocytes, and NK cells. All showed expression of TMPRSS13, with large differences in the percentage of positive cells between PBMCs from different donors, but with a relatively similar MFI of the corresponding TMPRSS13^+^ population. Based on the MFI, monocytes, B lymphocytes, and NK cells showed the highest level of TMPRSS13 expression. We differentiated macrophages from circulating monocytes and tested the expression of TMPRSS13 by surface IF. The level of expression was variable in these cells showing fine punctate staining at the cell surface and was confirmed by FCM (**Figure 5G**).

### TMPRSS13 facilitates the processing and fusogenic properties of SARS-CoV-2 spike

**Figure 6:**
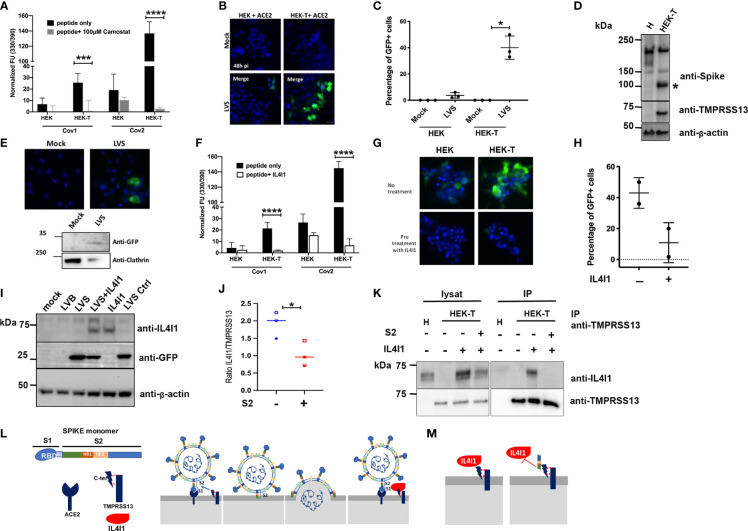
HEK cells expressing TMPRSS13 are infected by spike-pseudotyped virus and IL4I1 and spike compete for binding to TMPRSS13. **(A)** HEK or HEK-T cells were incubated at 37°C with either the fluorogenic peptide Cov1 (spike protein of the SARS-Cov) or Cov2 (spike protein of the SARS-CoV-2). For Camostat treatment, cells were preincubated for 2 h with 1 mM Camostat, followed by the addition of 100 μm during the test. Fluorescence due to peptide cleavage was periodically measured. End-point measurements are expressed as arbitrary fluorescence units (FU) after subtraction of the background (fluorescence measurements without peptide). Data are presented as the average ± SD of 2 to 3 experiments, each performed in triplicate. **(B, C)** HEK and HEK-T cells were transfected with a plasmid expressing ACE2. Forty-eight hours later, cells were infected for 48 h with a spike pseudotyped virus (LVS) expressing GFP. Cells were observed under a fluorescent microscope **(B)** and GFP positive cells quantified **(C)**. Data are from N =3 independent experiments. **(D)** HEK **(H)** or HEK-T (HT) cells transiently transfected for 48 h with a vector coding for spike protein were analyzed by WB using an anti-spike antibody followed by an anti-DYK antibody. Blots were re-hybridized with an anti-actin antibody as a loading control. * S2 fragment. **(E)** hMDM were differentiated from monocytes for six days, infected with the LVS or mock infected for 48 h, and observed under a fluorescence microscope (top panels) or analyzed by WB (bottom panels). GFP (top blot). Clathrin (lower blot) was used as a loading control. Representative images are shown. **(F)** HEK or HEK-T cells as in **(A)**, preincubated for 2 h with IL4I1. The test was performed as in **(A)**. Data are from N=2-3 independent experiments. **(G)** HEK or HEK-T cells were pre-incubated or not with IL4I1 and infected with LVS and followed by IF. **(H)** Quantification of experiments shown in **(G)**. **(I)** hMDM pre-incubated or not with IL4I1 and infected with LVS for 48 h were analyzed by WB for IL4I1 (top panel), GFP (to detect LVS) (center panel), and β-actin (lower panel). As control, a bald pseudotyped virus (LVB) was used. A representative blot is shown. **(J, K)** HEK and HEK-T cells pre-incubated with 20 µg **(J)** or 80 µg **(K)** of recombinant S2 fragment for 2 h before IL4I1 addition were co-immunoprecipitated as in **Figure 4**. **(J)** Quantified signals using ImageJ are expressed as the ratio of arbitrary units of IL4I1 and TMPRSS13. Data are from N=3 independent experiments. **(K)** Western-blot image of IL4I1/TMPRSS13 Co-IP performed in the presence of equivalent quantities (80 µg) of spike S2. **(L)** Schematic representation of the inhibition of the interaction of spike with TMPRSS13. Left: The spike protein interacts with ACE2 at the surface of target cells and TMPRSS13 cleaves the protein, liberating the S2 fragment, which changes conformation *via* the HR1 and HR2 domains, inserts into the host cell membrane, and favors envelope and cell membrane fusion. Right: if IL4I1 is present, it binds to TMPRSS13 and blocks it from interacting with the spike protein and cleaving it. **(M)** Schematic representation of the inhibition of the interaction of IL4I1 with TMPRSS13. Left: IL4I1 binds to TMPRSS13 on the plasma membrane of T lymphocytes and inhibits their activation. Right: The spike S2 fragment binds to TMPRSS13 and prevents IL4I1 from binding to the lymphocyte, possibly blocking its effect. Statistical analysis performed with a multiple t-test for panels A & F, and a two-tailed paired t-test for panels C & J, *p < 0.05, ***p < 0.001, and ****p < 0.0001.

TMPRSS13 belongs to the same family of serine proteases as TMPRSS2, which cleaves SARS-CoV-2 spike protein and facilitates envelope fusion with host cell membranes (15). TMPRSS13 is already known to facilitate the entry of influenza virus and coronavirus into host cells (22) and has been recently shown to cleave SARS-CoV-2 spike in cells ectopically expressing TMPRSS13 (17, 23, 24). We first verified the capacity of TMPRSS13 to cleave the S1/S2 fragment of SARS-CoV viruses using a fluorogenic peptide cleavage assay with the HEK and HEK-T cell lines (**Figure 6A**). Indeed, the expression of TMPRSS13 allowed the cleavage of the SARS-CoV peptide (3.9-fold higher signal than in non-expressing HEK cells) and more efficiently that of the SARS-CoV-2 peptide (7-fold increase). Digestion was blocked when the cells were pre-treated with Camostat, a known inhibitor of this family of serine proteases (15). To verify that TMPRSS13 expression facilitates the entry of SARS-Cov-2 GFP^+^ spike-pseudotyped lentivirus (LVS) into cells, we transfected HEK and HEK-T cells with a plasmid encoding the primary virus receptor ACE2 before infecting them with LVS. The presence of TMPRSS13 facilitated the entry of GFP^+^ LVS into HEK-T cells relative to HEK cells, resulting in 40 ± 8.9% and 3.7 ± 2.1% GFP^+^ cells at 48 hours post-infection, respectively (**Figures 6B, C**). This corresponded to a more important processing of the spike protein into its fusogenic S2 fragment (**Figure 6D**). Human macrophages, which naturally express TMPRSS13, could be also infected by the spike pseudotyped virus (**Figure 6E**).

### IL4I1 and SARS-CoV-2 spike compete for the interaction with TMPRSS13

As both IL4I1 and spike interact with TMPRSS13, we looked for homology between these proteins. The two proteins show a small N-terminal region of homology, corresponding to their signal peptide. We found no homology before the S1/S2 cleavage site of spike. On the contrary, we found the S2 fragment to share moderate homology with IL4I1 (**Figure S4**), which reached 24% identity and 57% similarity in some regions. Most of the similarity resided in the cleavage S1/S2 region, in the Heptad Region (HR)1, HR2 and in the fusion domains of spike. For the S1/S2 region, 5 amino acids were identical, 2 conserved, 2 semi-conserved with a gap of 6 amino acids that includes the cleavage point. For the 51 amino acids of HR1, 12 amino acids were identical, 9 conserved, 5 semi- conserved. For the 40 amino acids comprising the HR2, 6 were identical, 3 conserved and 9 semi-conserved. Finally, for the 39 amino acids of the fusion domains, 8 amino acids were identical, 10 conserved and 5 semi-conserved.

Considering this homology, we hypothesized that IL4I1 may interfere with the ability of TMPRSS13 to cleave spike. We thus preincubated HEK-T cells with recombinant IL4I1 prior to testing cleavage of the S1/S2 peptide (**Figure 6F**). Indeed, IL4I1 almost completely prevented SARS-CoV-2 peptide cleavage. Pre-incubation with recombinant IL4I1 also almost completely abolished the capacity of SARS-CoV-2 pseudovirus to infect HEK-T cells (**Figures 6G, H**). Moreover, the addition of IL4I1 to hMDM before infection with the SARS-CoV-2 LVS significantly decreased entry of the virus (**Figure 6I**). To verify if the spike S2 fragment might interfere with the interaction of IL4I1 with its receptor, we performed co-IPs of TMPRSS13 and IL4I1 in the presence of the S2 fragment of the spike protein (**Figures 6J, K**). When an excess of IL4I1 (20 µg of S2 per 80 µg of IL4I1) was used, the presence of the S2 fragment decreased IL4I1 binding to TMPRSS13 by approximately 50% (**Figure 6J**). When the amount of S2 was equivalent to that of IL4I1, the binding of IL4I1 to HEK-T cells was completely abolished (**Figure 6K**). Thus, IL4I1 can interfere with the interaction between spike and TMPRSS13 and reciprocally, the spike protein interferes with IL4I1 binding to its receptor.

## Discussion

This study provides two major findings. First, we show the existence of a new candidate receptor for the immunosuppressive enzyme IL4I1 and identify this protein as the serine protease TMPRSS13. This interaction may lead to intracellular signaling and may be important for IL4I1-mediated immunosuppression. Second, we have confirmed that TMPRSS13 facilitates spike cleavage and that spike pseudotyped virus can enter immune cells. Importantly, by interacting with TMPRSS13, IL4I1 and the spike protein can interfere with each other.

Although IL4I1 has several effects on the immune system, its mechanism of action remains partially elusive. In particular, the rapid effects on the TCR signaling pathway that have been observed (5) cannot be solely explained by the enzymatic activity of IL4I1, including the recently discovered AHR activation (11). The secretion of IL4I1 (3), and the observation that it can be found on the surface of T-cells after interaction with the cells that produce it (5), both suggested that IL4I1 can act as a cytokine. Here, using a combination of biochemical and immunological methods, we show that IL4I1 indeed binds to the transmembrane protein TMPRSS13, a serine protease recently shown expressed and important in human cancers and in viral infections (22, 25, 26). Both these proteins are enzymes but their reciprocal interaction indicate an additional role. Proteins with dual function have been described as “moonlighting” proteins (27). The precise physiological moonlighting function(s) of IL4I1 and TMPRSS13 acting as a ligand-receptor pair remains to be defined.

We show that TMPRSS13 is expressed by various immune cell types, with the percentage of positive cells greatly varying between donors, suggesting that the effect of IL4I1 could vary between individuals. Indeed, previously observed impact of IL4I1 on TCR signaling were donor dependent (5). The expression of TMPRSS13 by T and B lymphocytes may explain, at least partially, the action of IL4I1 on these cells. TMPRSS13 appears to be widely expressed by other circulating populations, suggesting that IL4I1 may have a larger spectrum of action in the immune system than currently known.

We observed that the C-terminal cleavage of TMPRSS13 is more prevalent and already present on primary T cells and cell lines where this protease may be already active. Such activation was not visible in primary B cells or in HEK-T cells overexpressing TMPRSS13. Serine transmembrane proteases show autocatalytic activity that is normally limited by Kunitz-type serine protease inhibitors, hepatocyte growth factor activator inhibitor (HAI)-1 or HAI-2 (19). T lymphocytes do not express these inhibitors and are often negative for other inhibitors, such as serpins (The Human Protein Atlas) (20). Their absence may explain the pre-activation of TMPRSS13 in T cells. The consequences of such activation are yet to be elucidated.

TMPRSS13 belongs to the same family of proteases as TMPRSS2, the protease involved in SARS-CoV-2 spike protein cleavage (15). The role of TMPRSS13 in the processing of envelope proteins was already described for influenza virus and has been recently shown for coronavirus (17, 23, 24). We confirm that TMPRSS13 can facilitate the entry of SARS-CoV-2 into cells that express it.

The interaction of IL4I1 and spike with TMPRSS13 led us to look for similarities between their sequences. Much to our surprise, the S2 fragment has several regions of similarity and/or homology with the human IL4I1 protein. These regions may be involved in the interaction of these proteins with TMPRSS13 and may indicate some convergent evolution (28).

The presence of IL4I1 blocked the digestion of spike and limited entry of the virus into the cells (see schema in **Figure 6L**). This should be important for the effects of SARS-CoV-2 on immune cells. Here, we show that macrophages can be infected by a pseudovirus bearing the spike protein. This is consistent with data from patients that indicate that both alveolar and monocyte-derived macrophages can be infected and modified by the virus (17, 29–33). As these cells are known to be resistant to viral infections but can harbor viruses, allow their replication, and behave as a Trojan horse (34), our observation could provide the basis for attractive anti-viral therapies to limit the consequences of immune cell infection.

Reciprocally, we have shown that the S2 fragment can block the interaction of IL4I1 with TMPRSS13 as depicted in **Figure 6M**. Such interference may be exploited to limit the immunosuppressive effect of IL4I1 in situations in which it is detrimental, such as cancer. Indeed, IL4I1 overexpression is frequent in the tumor microenvironment of cancer patients (7) and has been be correlated to a worsen outcome (35).

IL4I1 is an interferon (IFN)-responsive gene. Indeed, its expression is strongly induced by type I IFNs in myeloid cells (2). Moreover, IL4I1 is detected in IFNγ-rich inflammatory lesions, including tumors and SARS-CoV-2 infected tissues (7, 36). Due to immunosuppression induced both by tumor development and specific treatment, cancer patients are more susceptible to severe COVID-19 (37). However, patients who express high levels of IL4I1 in their tumor might benefit from a systemic effect of IL4I1 competing with spike, when contracting SARS-COV-2 infection. In one of the few studies testing for SARS-CoV-2 infection in cancer patients asymptomatic for COVID-19, a slightly lower incidence of SARS-CoV-2 was detected in comparison to that of the local community (38). However, both incidences were very low at the time of this study.

In conclusion, we have identified TMPRSS13 as a candidate surface receptor for the immunosuppressive enzyme IL4I1 and have shown that IL4I1 and the envelope protein spike of SARS-CoV-2 can interfere with each other for the interaction with TMPRSS13. Such interference open perspectives for the development of new treatments for COVID-19 or cancer patients.

## Materials and methods

### Reagents

TriCEPS-biotin, TriCEPS-TAMRA Transferrin, and Na periodate were purchased from Dualsystems Biotech AG (Schlieren – Switzerland). The Pan T-cell isolation kit (130–096–535) and the B cell isolation kit II (130-091-151) were purchased from Miltenyi Biotech (Paris, France). Recombinant human IL4I1 (5684-AO) and spike S2 fragment (10594-CV) were purchased from R&D, Biotechne (Noyal-Chatillon-sur-Seiche, France). TMPRSS13 DNA (NM_001077263.2), angiotensin converting enzyme 2 (ACE2) DNA (NM_021804) and Anti-DYK antibodies (A00187) were purchased from Genscript (Leiden, Netherlands). Anti-TMPRSS13 polyclonal antibodies (AP14675b) and Protein A/G agarose beads (Sc2003) of Santa Cruz Biotechnology were purchased from Cliniscience (Nanterre, France). The anti-TMPRSS13 N-terminal antibody (Ab59862) was purchased from Abcam (Paris, France). All culture reagents were purchased from Life Technologies. Anti-TMPRSS13 antibody (Pa5-30935), Texas-red phalloidin (T7471), goat anti-rabbit-Alexa Flour 488 (A21206), goat anti-rabbit AlexaFlour 647 (A21244) antibodies, poly D-lysine (A389040), ProLong™ Gold Antifade Mounting Media (P10144), Lipofectamine 2000 (11668027), eBioscience Fixable Viability Dye-efluor 450 (65-0863), anti-human CD4 APC-eFlour 780 (47-0049-42, clone RPA-T4), anti-human CD28 (16-0289-81, clone 28.2), and their respective isotype controls were purchased from ThermoFisher (Villebon-sur-Yvette, France). Anti-human CD14-FITC (555397, clone M5E2), anti-human CD3-PE (555333, clone UCHT1), and their respective isotype controls were purchased from BD Biosciences (Pont de Claix, France). Anti-human CD56-PC5.5 (A79388, clone N901), anti-human CD19-PC7 (IM3628, clone J3-119), and their respective isotype controls were purchased from Beckman Coulter (Villepinte, France). Anti-human CD4-BV510 (BLE300546, clone RPA-T4), anti-human CD8 BV711 (BLE301044, clone RPA-T8), their respective isotype controls, True Staining Monocyte blocker (42610), and PE-streptavidin (405203) were purchased from Biolegend (Amsterdam, Netherland). Goat-anti-rabbit-HRP (7074) and horse-anti-mouse-HRP (7076) antibodies from Cell Signaling were purchased from Ozyme (Saint-Cyr-L’École, France). Pre-cast SDS-PAGE gels, Precision Plus Protein™ Dual Color Standards (1610374) and TidyBlot Western Blot Detection Reagent-HRP (STAR209) were purchased from BioRad (Marne-la-Coquette, France). Gel running buffer (TG-SDS EU0510) and transfer buffer (TG EU0550) were purchased from Euromedex (Souffelweyersheim, France). Roche cOmplete™ Mini Protease Inhibitor Cocktail (4693124001), anti-myc monoclonal antibodies (M4439, clone 9E10), Millipore Immobilon-P PVDF membranes (IPVH00010), Millipore Luminata Crescendo, Amicon ultra 4 30K (UFC803008), and the Duolink PLA kit (DU092007) were purchased from Merck Millipore (Guyancourt, France). Monoclonal anti-rabbit antibodies against IL4I1 were produced against a peptide by our laboratory (clone 43-7, patent EP18306563.0). Concentrated 32% Paraformaldehyde (PFA) was purchased from Electron Microscopy Sciences (Biovalley, France) and diluted in PBS at a final concentration of 4%.

### Cells

Jurkat cells were cultivated in RPM1 1640 containing 10% fetal calf serum (FCS), 100 units/ml penicillin, and 100 μg/ml streptomycin. A549 cells expressing TMPRSS2 were a kind gift from P-Y Lozach. Human embryonic Kidney (HEK) cells were cultivated in DMEM containing 10% FCS, 100 units/ml penicillin, and 100 μg/ml streptomycin. HEK cells expressing inducible recombinant human IL4I1 (rhIL4I1) have been described elsewhere (3) and were used in certain experiments (PLA and co-IP). For these experiments, IL4I1 was induced using 2 mg/ml doxycycline for a week from sub-confluent cells. Over the last 16 h, the recombinant protein was recovered in PBS containing Ca^++^ and Mg^++^ and subsequently concentrated using an Amicon filter with a cut-off of 30 kDa. After concentration, the IL4I1 activity and protein content were measured. A volume of the conditioned PBS corresponding to 10,000 U of specific activity (pmol H_2_O_2_/h/mL IL4I1) was added to a well containing the cells in a six-well plate.

Peripheral blood mononuclear cells (PBMCs) were obtained by cytapheresis from healthy donors from the French Blood bank (Etablissement Français du sang, EFS) and isolated using a Ficoll density gradient. Monocyte-derived macrophages (hMDMs) were differentiated from PBMCs according to the method described in Lê-Bury et al. (39) for six days. CD3^+^ cells and naïve B cells were isolated from donor PBMCs using the magnetic bead Pan T isolation kit and B Cell Isolation Kit II from Miltenyi respectively, according to the manufacturer’s instructions.

### Pseudovirus

EGFP-expressing pseudovirus (LVS) based on a pLV[Exp]-CMV>EGFP lentivirus and typed with spike protein or bald controls (LVB) were purchased from Vectorbuilder Gmb. For the infections, cells plated in six-well plates (transfected HEK or hMDM) were washed with PBS followed by the addition of 500 µL medium without antibiotics containing the diluted virus and incubation for 1 h at room temperature out under gentle agitation. Plates were washed with medium and fresh complete medium was added for the rest of the experiment.

### DNA

The sequence of TMPRSS13 (NM_001077263.2) was cloned into a pcDNA3.1(+)-C-DYK vector and that of ACE2 (NM_021804) into a pcDNA3.1(+)-C-HA vector. All DNA constructs were produced by Genscript (Leiden, Netherland).

### Transfections

Transfection of HEK cells with TMPRSS13 DNA was performed using Lipofectamine 2000 according to the manufacturer’s instructions. For stable transfectants, cells were diluted the day after transfection and the following day selection started using 1.2 mg/mL G418. The selection media was changed every two days until the appearance of antibiotic-resistant cell colonies. Each colony was then isolated and verified for protein expression by WB. One clone was chosen for the rest of the experiments (HEK-T6).

### TriCEPS experiments

Ligand receptor-based capture (LRC) and mass spectrometry was performed using LRC-TriCEPS-biotin (Dualsystems Biotech), as previously described (18). Briefly, capture was performed on human CD3^+^ T cells or Jurkat cells using hrIL4I1 bound to the LRC-TriCEPS bait. Anti-human CD28 antibody or transferrin were used as positive control baits and glycine as a negative control. For LRC-TriCEPS binding, 240 μg of control bait or rhIL4I1 protein were buffer exchanged to 150 μl 25 mM HEPES, pH 6.5. LRC-TriCEPS V.3 was added to each reaction and the reaction mixed and incubated at 22°C with gentle shaking for 90 min. After the reaction, the bound baits were quenched for 30 min on ice with an equal volume of a 125 mM Tris-HCl solution, pH 6.5.

For FCM binding experiments, 0.5 x 10^6^ cells (Jurkat or purified CD3^+^ lymphocytes) were labeled with Fixable Viability Dye efluor 450 for 30 min at room temperature. Cells were then incubated with 1.2 μg anti-CD28 antibodies (CD3^+^ cells), transferrin (Jurkat cells), or hIL4I1 previously bound to LRC-TriCEPS (TriCEPS-conjugated) for 1 h on ice. After extensive washing, LRC-TriCEPS-baits bound to cells were stained with streptavidin-PE and CD4 positive lymphocytes identified using an anti-CD4-APC-eFlour 780 antibody and analyzed on a LSRII flow cytometer (BD Bioscience, France). Analysis was performed using Flowjo software.

For mass spectrometry identification of interacting proteins, 1.2 x 10^8^ Jurkat cells were oxidized by treatment with 1.5 mM sodium metaperiodate at 4°C for 15 min. After oxidation, the cells were washed, divided into two parts, and incubated with TriCEPS-transferrin or TriCEPS-rhIL4I1 (see above) for 90 min in the dark under rotation. For each condition, cells were divided into three tubes and frozen at -80°C. Mass spectrometry analysis was performed using the Dualsystem approach according to Frei et al. (18). Samples were analyzed on a Thermo LTQ Orbitrap XL spectrometer fitted with an electrospray ion source. Tryptic peptides were measured in data dependent acquisition mode (TOPN) in a 120 min gradient using a 10-cm C18 packed column. Progenesis software was used for raw file alignment and feature detection, the Comet Search Engine for spectra identification, and the Trans-Proteomic Pipeline for statistical validation of putative identifications and protein inference.

Upon protein inference, relative quantification of the control and ligand samples was performed based on the ion extracted intensity and differential protein abundance was tested using a statistical ANOVA model, followed by a multiple testing correction. This model assumes that the measurement error follows a Gaussian distribution and views individual features as replicates of a protein’s abundance and explicitly accounts for such redundancy. It tests each protein for differential abundance in all pairwise comparisons of ligand and control samples and reports the p-values. The Uniprot human proteome data base was used for analysis.

For TAMRA-TriCEPS experiments, Jurkat cells were incubated with TAMRA-TriCEPS Transferrin or TAMRA-TriCEPS-rhIL4I1 for 1 h on ice in the dark. Following incubation, cells were washed and resuspended in PBS containing 0.1% FCS and 6.5 x 10^4^ were spun onto 12-mm glass coverslips previously coated with 0.1% poly-D-lysine. Cells were then fixed with 4% para-formaldehyde (PFA) for 20 min and the coverslips mounted with Prolong containing DAPI. Cells were observed using a fluorescence Axioimager M2 EC (Zeiss, France) with Plan Neofluar 40X/0.75 and x63/1.25 objectives.

### Immunofluorescence

HEK cells were grown on glass coverslips pre-coated with 0.1% poly-D-lysine. hMDMs were cultivated and differentiated on glass coverslips. Cells were fixed with 4% PFA for 20 min at RT. For hMDMs, 1 µL of True Stain monocyte blocker was added for 10 min before labeling to each coverslip to block FC receptors. For non-permeabilized cells, coverslips were directly blocked with 10% bovine serum albumin (BSA) followed by incubation with primary and secondary antibodies. For permeabilized cells, the cells were incubated for 4 min at room temperature in a 0.1% Triton X100 solution before being blocked with BSA. After staining, coverslips were mounted using Prolong containing DAPI and observed using a fluorescence Axioimager M2 EC using Plan Neofluar 40X/0.75 and x63/1.25 objectives.

### Proximity ligation assay (PLA)

PLA was performed according to the manufacturer’s instructions. Briefly, HEK or HEK-T expressing TMPRSS13 were plated onto 0.1% poly-D-lysine-treated coverslips in 24-well plates the day before the assay. Cells were incubated for 1 h on ice with conditioned PBS containing rhIL4I. After extensive washing with PBS, cells were fixed with 4% PFA for 20 min, blocked, and incubated with mouse monoclonal anti-DYK and rabbit monoclonal anti-IL4I1 antibodies for 30 min at 37°C. Cells were then incubated with the secondary antibodies bound to the oligonucleotide probes for 1 h at 37°C. Following several washes, a solution containing DNA ligase was added to the samples, followed by a 30 min incubation at 37°C. Finally, washed samples were incubated in an amplification solution containing polymerase and a Cy3 fluorescent nucleotide. Coverslips were then washed and mounted with Prolong containing DAPI and observed under a fluorescence microscope with a 20X Acroplan x20/0.45 objective. For PLA experiments with Jurkat cells, mouse monoclonal anti-myc and rabbit polyclonal anti-TMPRSS13 antibodies were used.

### Co-immunoprecipitation

HEK or HEK-T cells from a six-well plate were incubated for 1 h on ice with concentrated hIL4I (10000U/well). After 10 washes with 1 ml PBS, cells were lysed in 500 µL lysis buffer (50 mM Tris HCl, pH 7.5, 150 mM NaCl, 2 mM EDTA, 0.5% Triton X100) containing cOmplete™ Protease Inhibitor Cocktail. Lysates were spun at 5,000 x g for 5 min and whole cell supernatants incubated overnight with rotation at 4°C with 1.5 µg anti-DYK antibodies or 3 µg anti-IL4I1 antibodies (clone 43-7, patent EP18306563). After the addition of 50 µL of a 50% suspension of protein A/G agarose beads and incubation for an additional 2 hat 4°C, the agarose beads were washed three times with 1 mL lysis buffer and resuspended in 50 µL Laemmli sample buffer (0.1 M Tris-HCl, pH 6.8, 10% glycerol, 1% SDS, 0.05 M DTT, 0.2% bromophenol blue). After an incubation of 5 min at 95°C and samples were loaded onto a 10% SDS-PAGE. Ten microliters of the whole-cell supernatants were run in parallel as input controls. For the experiments with the S2 fragment of the spike protein, 20 or 80 µg of the recombinant protein were added on the cultures 2 h before the addition of IL4I1 as described above.

### Flow cytometry

Viable PBMCs were identified using Fixable Viability Dye efluor 450. Then, surface labeling was performed (anti-human CD3-PE, CD4-BV510, CD8-BV711, CD14-FITC, CD19-PC7, and CD56-PC5.5) by adding the antibody cocktail containing True Stain monocyte blocker on ice and performing all the steps at 4°C. After washing, PBMCs were fixed with 4% PFA. For Jurkat cells and HEK-T cells, the cells were directly fixed. The fixed cells were washed in PBS and resuspended in PBS containing 1% FCS and 10 ng/ml of the anti-TMPRSS13 antibody and incubated for 30 min at 4°C, followed by incubation with goat ant-rabbit Alexa Fluor 647 antibody. Data was collected using a Fortessa X20 flow cytometer (BD Bioscience, France) and analyzed using FlowJo software.

### Western blots

Proteins separated by SDS-PAGE (pre-cast gels NuPAGE Thermo Scientific or BioRad) were transferred to PVDF membranes (Merck Millipore, France) at 100 mV for 2 h or overnight at 30 mV in 25 mM Tris, 192 mM glycine, and 20% (v/v) ethanol. Membranes were blocked with a 10% BSA solution in TBS-T (50 mM Tris-Cl, pH 7.6, 150 mM NaCl, 0.05% Tween-20) before incubation with specific antibodies diluted in 1% BSA-TBST. Primary antibodies were revealed using anti-rabbit-HRP, anti-mouse-HRP antibodies or TidyBlot Western Blot Detection Reagent-HRP using Luminata Crescendo (Merck-Millipore, France) or Pierce Dura. Blots were re-probed after HRP inactivation by incubation in 30% H_2_O_2_ (8.8 M) for 30 min at 37°C according to Sennepin et al. (40). Images were captured using a CCD camera (Autochemi system, UVP, UK) or Fusion FX devices (Vilber, France) and analyzed using ImageJ.

### IL4I1 activity

IL4I1 LAAO activity was measured against Phe as previously described (7).

### Spike *in-vitro* digestion system

The digestion test was performed according to Jaimes et al. (41). Peptides corresponding to the SARS-CoV and SARS-CoV-2 spike (S) S1/S2 sites composed of the sequences HTVSLLRSTSQ and TNSPRRARSVA, respectively, and harboring the (7-methoxycoumarin-4-yl)acetyl/2,4-dinitrophenyl (MCA/DNP) FRET pair were synthesized by Biomatik (Wilmington, DE, USA). Briefly, HEK and HEK-T cells were seeded at 10^4^ cells/well in a flat bottom 96-well plate the day before the experiment. Cells were washed with 200 µL PBS and then incubated with 200 µM of each peptide. For certain experiments, cells were preincubated for 2 h with 1 mM Camostat or 1500U/well concentrated recombinant IL4I1. Camostat was maintained at 100 μM during the assay. Plates were placed in a Varioskan™ LUX plate reader and fluorescence emission at 37°C was kinetically recorded using λex 330 nm and λem390 nm wavelengths for up to 45 min. Data are shown as the mean values for the endpoints of triplicates from which the background values (fluorescence of PBS only) were subtracted.

### Protein alignment

The Spike (sp|P0DTC2|SPIKE_SARS2) and IL4I1 protein sequences (NP_690863.1) were aligned using Clustal Omega and analyzed using Jalview (42). Coloring of residues is performed by annotation with a 7 threshold and at 50% conservation.

### Statistical analysis

Values are expressed as the mean ± SEM where applicable. Statistics were performed as specified in the figure legends, using Graphpad Prism 9, except for **Figure 2** (see above TriCEPS experiments). Paired or unpaired t tests were used depending on inter-experiment variation of the values. *p < 0.05, **p < 0.01, ***p < 0.001, and ****p < 0.0001.

## Data availability statement

The original contributions presented in the study are included in the article/**Supplementary Material**. Further inquiries can be directed to the corresponding authors.

## Author contributions

Designed experiments: FC, FN, and VM-F. Performed experiments: JG, CN, AD, M-LP, FN, and FC. Analyzed the results: FC, FN, VM-F, JG, JC, and AD. Funding acquisition: FC, FN, VM-F, and JC. Wrote the manuscript - original draft: FC, VM-F, and FN. Wrote the manuscript – review & editing: FC, VMF, FN, and JC. All authors contributed to the article and approved the submitted version.

## Funding

This work was supported by Bristol-Myers Squibb Foundation for Research in Immuno-Oncology (FC), Erganeo Pre-maturation grant (FC). INSERM, the CNRS, and Université Paris Cité (FN laboratory); INSERM and Université Paris Est-Créteil (UPEC) (JC laboratory).

## Acknowledgments

We thank the IMRB cytometry and imaging facilities as well the IMAG’IC platform (Institut Cochin) which is a member of the National Infrastructure France BioImaging (ANR-10-INBS 04). The manuscript has been professionally corrected by a native English speaker from the scientific editing and translation company, Alex Edelman & Associates.

## Conflict of interest

The authors declare that the research was conducted in the absence of any commercial or financial relationships that could be construed as a potential conflict of interest.

## Publisher’s note

All claims expressed in this article are solely those of the authors and do not necessarily represent those of their affiliated organizations, or those of the publisher, the editors and the reviewers. Any product that may be evaluated in this article, or claim that may be made by its manufacturer, is not guaranteed or endorsed by the publisher.
