# Supplementary material for: IL4I1 binds to TMPRSS13 and competes with SARS-CoV-2 spike
Source: Front Immunol. 2022 Sep 5;13:982839. doi: 10.3389/fimmu.2022.982839 (PMC9483092; doi:10.3389/fimmu.2022.982839)
Supplement: Supplementary file 1 [file DataSheet_1.docx]

Supplementary material for

IL4I1 binds to TMPRSS13 and competes with SARS-Cov2 Spike

**Authors:** Jérôme Gatineau,^1^, Charlotte Nidercorne ^2&^, Aurélie Dupont ^1^,Marie-Line Puiffe^1^, José L Cohen ^1,3^ , Valérie Molinier-Frenkel ^1,4^ *, Florence Niedergang ^2^*, and Flavia Castellano^1,5^*

^1^Univ Paris Est Creteil, INSERM, IMRB, F-94010 Creteil, France

^2^ Université Paris Cité, CNRS, INSERM, Institut Cochin, F-75014 Paris, France

^3^AP-HP, Hopital H Mondor, CIC Biotherapies, Créteil 94010, France

^4^AP-HP, Hopital Henri Mondor, Departement d’Hematologie-Immunologie, Créteil 94010, France

^5^AP-HP, Hopital Henri Mondor, Plateforme des Ressources Biologiques, Créteil 94010, France

^&^ Present address: ^1^Univ Paris Est Creteil, INSERM, IMRB, F-94010 Creteil, France

Correspondence to:

Flavia Castellano, flavia.castellano@inserm.fr,

Florence Niedergang, florence.niedergang@inserm.fr

Valérie Molinier-Frenkel, valerie.frenkel@inserm.fr

**Fig. S1**. Gating strategy for TriCEPS labeling. Lymphocytes were identified on the SSC-A FSC-A plot. After the exclusion of doublets, cells were gated on Viability Dye (VD)-negative cells. For Jurkat cells, the LCR-TriCEPS labeling was measured in this window. For lymphocytes, the LCR-TriCEPS labeling a was further analyzed on the CD4^+^ population.

**Fig. S2. Validation of the HEK clone expressing TMPRSS13 and the commercial anti-TMPRSS13 antibody.** HEK cells stably transfected with human DYK-tagged TMPRSS13 cDNA were analyzed by WB using an anti-DYK antibody (**A**). HEK clone HT6, expressing TMPRSS13, was selected for further studies. Clone HEK-T6 (**B**) and transiently transfected cells (**C**) were tested using a commercially available anti-TMPRSS13 antibody directed against the C-terminal part of TMPRSS13 by WB. (**D**) A549 cells expressing or not TMPRSS2 were tested by WB using the anti-TMPRSS13 antibody directed against the C-terminal part of TMPRSS13. (**E**) HEK cells and HEK cells overexpressing TMPRSS13 (HEK-T) were tested by FCM using the anti-TMPRSS13 antibody. Filled grey plot: non-labelled cells, black line: HEK cells, red line: HEK-T cells. **(F**) Triton X100 permeabilized HEK (top) and HEK-T (bottom) cells were labeled with an anti-DYK antibody followed by an anti-mouse Alexa488 antibody. **(G**) Triton X100 permeabilized HEK (top) and HEK-T (bottom) cells were labeled with an anti-TMPRSS13 rabbit polyclonal antibody, followed by an anti-rabbit Alexa 488 antibody. Texas red phalloidin: actin cytoskeleton and DAPI: nuclei. Bar = 10 μM. (**H**) Non-permeabilized HEK (top) and HEK-T cells (bottom) were labeled with an anti-DYK antibody, followed by an anti-mouse Alexa 488 antibody. (**I**) Non-permeabilized HEK (top) and HEK-T cells (bottom) were labeled with an anti-TMPRSS13 antibody, followed by an anti-rabbit Alexa 488 antibody. DAPI : nuclei. Bar = 10 μM.

**Fig. S3.** **Proximity ligation assay of Jurkat cells incubated with recombinant IL4I1 reveals spatial proximity of IL4I1 and TMPRSS13**. Jurkat cells were incubated with recombinant hIL4I1 at 4°C for 1 h. After extensive washing and 4% PFA fixation, cells were incubated with rabbit anti-TMPRSS13 and/or mouse anti-myc primary antibodies and secondary anti-mouse and anti-rabbit antibodies bound to specific primers. After ligation and the PCR reaction containing a red fluorescent nucleotide, the coverslips were mounted with DAPI to stain the nuclei. (**A**) No primary antibodies, (**B**) primary anti-DYK antibody only, (**C**) Jurkat cells without incubation with IL4I1 and incubated with primary and secondary antibodies, **(D**) Jurkat cells incubated with IL4I1 and the primary and secondary antibodies. Images were captured using an Axioimager 2 fluorescent microscope and analyzed using ImageJ. Representative images from three independent experiments (n = 3). Bar = 10 μM.

**Fig. S4.** **Protein alignment of Spike and IL4I1**. The Spike (sp|P0DTC2|SPIKE_SARS2) and IL4I1 protein sequences (NP_690863.1) were aligned using Clustal Omega and analyzed using Jalview (*37*). Coloring of residues is performed with a 7 threshold and at 50% conservation. Boxes show the cleavage site, the fusion peptide (Fus1 &2), the Heptad Region (HR)1 and HR2 of the spike protein.
